# Protective efficacy of malaria case management for preventing malaria mortality in children: a systematic review for the Lives Saved Tool

**DOI:** 10.1186/1471-2458-11-S3-S14

**Published:** 2011-04-13

**Authors:** Julie Thwing, Thomas P Eisele, Richard W Steketee

**Affiliations:** 1 Malaria Branch, Malaria Branch, Center for Global Health, Centers for Disease Control and Prevention, Atlanta GA, USA; 2 Department of International Health and Development, Tulane University School of Public Health and Tropical Medicine, New Orleans LA, USA; 3 Malaria Control and Evaluation Partnership in Africa (MACEPA), a program at PATH, Batiment Avant Centre, 01210 Ferney-Voltaire, France

## Abstract

**Background:**

The Lives Saved Tool (LiST) model was developed to estimate the impact of the scale-up of child survival interventions on child mortality. New advances in antimalarials have improved their efficacy of treating uncomplicated and severe malaria. Artemisinin-based combination therapies (ACTs) for uncomplicated *Plasmodium falciparum* malaria and parenteral or rectal artemisinin or quinine for severe malaria syndromes have been shown to be very effective for the treatment of malaria in children. These interventions are now being considered for inclusion in the LiST model. However, for obvious ethical reasons, their protective efficacy (PE) compared to placebo is unknown and their impact on reducing malaria-attributable mortality has not been quantified.

**Methods:**

We performed systematic literature reviews of published studies in *P. falciparum* endemic settings to determine the protective efficacy (PE) of ACT treatment against malaria deaths among children with uncomplicated malaria, as well as the PE of effective case management including parenteral quinine against malaria deaths among all hospitalized children. As no randomized placebo-controlled trials of malaria treatment have been conducted, we used multiple data sources to ascertain estimates of PE, including a previously performed Delphi estimate for treatment of uncomplicated malaria.

**Results:**

Based on multiple data sources, we estimate the PE of ACT treatment of uncomplicated *P. falciparum* malaria on reducing malaria mortality in children 1–23 months to be 99% (range: 94-100%), and in children 24-59 months to be 97% (range: 86-99%). We estimate the PE of treatment of severe *P. falciparum* malaria with effective case management including intravenous quinine on reducing malaria mortality in children 1-59 months to be 82% (range: 63-94%) compared to no treatment.

**Conclusions:**

This systematic review quantifies the PE of ACT used for treating uncomplicated malaria and effective case management including parenteral quinine for treating severe *P. falciparum* malaria for preventing malaria mortality in children <5. These data will be used in the Lives Saved Tool (LiST) model for estimating the impact of scaling-up these interventions against malaria. However, in order to estimate the reduction in child mortality due to scale-up of these interventions, it is imperative to develop standardized indicators to measure population coverage of these interventions.

## Background

Malaria remains a serious global public health problem, with nearly one-third of the world population exposed to stable *Plasmodium falciparum* transmission, the species responsible for most malaria deaths [1]. WHO has estimated that 243 million malaria cases and 863,000 malaria deaths occurred globally in 2009, with over 90% occurring in sub-Saharan Africa [2].

Artemisinin combination therapies (ACT) are highly effective for preventing an uncomplicated *P. falciparum* malaria fever from progressing to severe disease, which can result in death [3,4]. Nearly all endemic countries have now adopted ACT as the first-line treatment for *P. falciparum* malaria [5]. Among children with severe malaria, effective case management with parenteral or rectal artemisinin or quinine is also recognized to be highly effective at preventing death [6-8]. More recently, a large scale trial of intravenous artemisinin has been shown to reduce mortality compared to quinine in African children with severe malaria [9]. However, the impact of prompt and effective treatment of uncomplicated malaria and case management of severe malaria on reducing child malaria deaths has not been quantified with placebo-randomized controlled trials for obvious ethical reasons.

In the absence of sufficient systems to monitor real-time trends in cause-specific child mortality in many developing countries, mathematical models are increasingly being used to estimate the impact of the scale-up of child survival interventions on child mortality. The Lives Saved Tool (LiST) is one such model developed by WHO and UNICEF’s Child Health Epidemiology Reference Group (CHERG) and based on the earlier work on effectiveness of interventions [10]. The model estimates child deaths prevented (within specific cause of death categories) due to intervention scale-up within a specified country as a function of three primary parameters: 1) the number of child deaths by cause projected to occur in each year (including population growth parameters over time); 2) the protective efficacy (PE) on cause-specific mortality (PE = 1 - relative risk (RR) * 100) for each intervention being scaled-up; and 3) increases in population coverage of each intervention [11].

We performed systematic literature reviews to identify studies that could be used to estimate the effect of prompt effective treatment of uncomplicated malaria and effective case management of severe malaria for preventing post-neonatal child malaria deaths, defined here as mortality directly following a clinical episode of malaria (within a 28 day follow up period for uncomplicated and during hospitalization for severe) in children 1-59 months (assuming no malaria deaths occur or could be accurately attributed during the neonatal period). We chose to focus on sub-Saharan Africa, given the vast majority of childhood deaths due to malaria occur there and as results obtained in this zone of primarily stable transmission may not apply to zones of unstable transmission. Results for the systematic reviews will be considered for default values for PE for the interventions to be included in the LiST model.

## Methods

These intervention effectiveness reviews were shaped in large part by the needs of the LiST model, as presented elsewhere [11]. In the LiST model, increases in coverage of an intervention result in a reduction of one or more cause-specific deaths or in reduction of a risk factor for death. Therefore the reviews and the grade process were designed to develop estimates of the effect of an intervention in reducing a death due to specific cause. A set of rules were also established and followed for generating estimated intervention effects for use in LiST, as presented elsewhere [11].

To quantify the PE of case management of uncomplicated and severe malaria for preventing malaria deaths in children, we conducted a systematic literature review to identify randomized controlled trials using PubMed. Studies were limited to those implemented in areas of endemic *P. falciparum* malaria transmission. Where sufficient data from individual or community-randomized controlled trials could not be identified, we considered all available evidence, including observational and Delphi studies, in quantifying the PE of interventions reviewed here. Where possible, study quality was assessed according to the GRADE technique [12], adapted by the Child Health Epidemiology Reference Group as outlined elsewhere [11]. We assessed the quality of the data sources contributing to each CFR estimate as high, moderate, or low (Table 1).

**Table 1:** Quality of sources of data for estimates

	Data source	Number of studies	Data source quality
Uncomplicated malaria - untreated	Delphi method	1	low
Uncomplicated malaria - treated	Deaths reported in clinical trials of ACTs for uncomplicated malaria	49	high
All hospitalized malaria - untreated	Mortality when treated with CQ in setting of high CQ resistance – hospital observational studies	2	low
All hospitalized malaria - treated	Mortality in hospitals offering high standards of care - hospital observational studies	4	moderate

A malaria death is defined as one death per parasitologically diagnosed clinical episode. It is recognized that non-lethal episodes of malaria frequently result in increased vulnerability to other infectious diseases and subsequent death due to another cause. Some have suggested that the likelihood of dying in the 8 weeks following discharge from a malaria diagnosis is equal to the likelihood of dying in the hospital [13]. However, lack of sufficient data precluded the inclusion of such deaths due indirectly to a *P. falciparum* infection.

### Prompt treatment of uncomplicated malaria with effective antimalarials

We sought to measure the PE of prompt ACT treatment of uncomplicated malaria for preventing child malaria mortality, as compared with no treatment, in *P. falciparum* malaria endemic settings. Uncomplicated malaria was defined as illness, febrile or otherwise, for which treatment was sought, with confirmation of a *P. falciparum* infection, either by microscopy or rapid diagnostic test. Given the absence of placebo-controlled trials of treatment of uncomplicated malaria, we adopted the following approach to estimate a relative risk for ACT versus no treatment of uncomplicated malaria for preventing malaria mortality, which requires the rate of malaria death (observed) in those treated with ACT and the rate of death in those untreated (natural history). A comprehensive and thorough review of comparative effectiveness studies of ACTs against other antimalarials for uncomplicated malaria was published in 2009 [3]. We retrieved the articles included in this review and abstracted mortality from each trial to determine case fatality ratios (CFR) despite prompt and effective treatment of uncomplicated malaria. The mean CFR across all trials used in this analysis was weighted by the sample size reported in each study. While literature searches to determine natural history of untreated uncomplicated falciparum malaria in children were fruitless, Sudre and Breman conducted a study using the Delphi method to garner expert opinion on the proportion of otherwise healthy children who would die from uncomplicated malaria if left untreated, within an area of stable *P. falciparum* transmission in Africa [14]. They found that experts estimated mortality in untreated children <2 years old with uncomplicated *P. falciparum* malaria to be 5%, and in untreated 2-5 year olds to be 2%. We used these estimates as proxies for mortality of untreated uncomplicated malaria (natural history). We estimated uncertainty using a sensitivity analysis. For the lower bound, the CFR among treated children was assumed double the observed estimate and the CFR among untreated children was assumed to be half the Delphi estimate. For the upper bound, the CFR among treated children was assumed half the observed estimate and the CFR among untreated children was assumed to be double the Delphi estimate.

### Effective case management of children hospitalized with malaria

We sought to estimate the PE of effective case management with intravenous quinine of children hospitalized with malaria for preventing child malaria mortality, as compared with no treatment, in sub-Saharan Africa. While severe disease treatment trials often focus on a restricted spectrum of severity, (e.g. only including cerebral malaria), in practice, children hospitalized with malaria demonstrate a variety of syndromes, including prostration, intractible vomiting, respiratory distress, severe malarial anemia, hypoglycaemia (often iatrogenic), multisystem organ failure, and cerebral malaria. A stringent definition of severe malaria is often applied for research purposes, but for programmatic uses, such as the LiST model, data regarding hospitalization and outcomes are more likely to be available for all hospitalized children. Thus we attempted to calculate protective efficacy of case management with intravenous quinine for all children hospitalized with malaria, for greatest programmatic utility.

We used observational trials and retrospective data reviews to examine treated and untreated mortality for all children hospitalized with malaria. We searched PubMed using the terms “severe malaria” and “mortality” to search for studies on mortality due to malaria in hospitalized African children to attempt to ascertain the CFR of malaria in this setting. Any hospital-based study conducted in a zone of stable transmission in sub-Saharan Africa that reported the proportion of children admitted with a diagnosis of malaria that had a fatal outcome was included.

From each report, we abstracted definitions of severity, quality of care, antimalarial used, age range, country, urban or rural environment, number of children included, and number of deaths. For studies in which one or more categories of the above were documented, we abstracted each separately. Studies that reported the outcome of all hospitalized children and offered care rated moderate to good were included in the estimate of CFR for hospitalized children with effective case management with intravenous quinine.

There are no randomized-placebo controlled trials to determine mortality reduction due to effective treatment of hospitalized children. As a proxy for the natural history of mortality of children eligible for hospitalization with malaria, we used mortality of children hospitalized with malaria in the setting of chloroquine failure, reported in two retrospective reviews retrieved through the above search. We recognize this will likely result in an underestimation of the natural history of mortality in children who went untreated for malaria requiring hospitalization, which would result in an underestimation of the protective efficacy of effective case management of severe malaria.

PE was calculated using the weighted mean CFR of children treated with effective case management with intravenous quinine, and the average of the two observational studies in the setting of chloroquine failure (we did not weight this due to the unbalanced sample sizes) for the natural history. For calculation of uncertainty surrounding protective efficacy, we conducted a sensitivity analysis, using the 95% confidence limits of the weighted mean CFR as upper and lower bounds in children with effective case management, and the range of CFR in the two retrospective studies as the upper and lower bounds of mortality in the setting of chloroquine failure.

## Results

### Treatment of uncomplicated malaria with effective antimalarials

Among the 49 double-blind randomized placebo controlled equivalency trials identified from our review [15-63], the weighted mean CFR among children with uncomplicated *P. falciparum* malaria treated promptly with an ACT was 0.068% (11 / 16,086) (95% CI .024-.112); an additional 67 cases were reported as early treatment failures that received rescue therapy and went on to recovery. These trials largely did not report the timing or other details for each death. This was used as the mortality in children receiving the intervention of prompt treatment with ACT. The estimates of 5% mortality and 2% mortality for untreated children <2 and 2-5 years old, respectively, were used as the proxy for the natural history of mortality in untreated children with uncomplicated *P. falciparum* malaria. Based on these findings, the PE of prompt treatment of uncomplicated *P. falciparum* malaria with ACT was estimated to be 99% (range 94 – 100%) for children <2 years old and 97% (range 86 – 99%) for children 2-5 years old (Table 2).

**Table 2:** Estimates of protective efficacy of prompt ACT treatment for uncomplicated *P. falciparum* malaria

Child age	**CFR among children receiving no treatment (per 10,000)**[14]	Estimated CFR among children receiving prompt ACT treatment (per 10,000)	Protective efficacy	Lower bound	Upper bound
**<2 years**	500	6.8	98.6%	94.4%	99.7%
**2-5 years**	200	6.8	96.6%	86.0%	99.3%

CFR: case fatality ratio

Protective efficacy = (1-Relative risk) * 100, where Relative risk = CFR receiving ACT / CFR no treatment

Lower bound based on sensitivity analysis where CFR among treated children assumed double (14 per 10,000) and CFR among untreated children assumed to be half (250 and 100 per 10,000 for children <2 and 2-5 years old, respectively).

Upper bound based on sensitivity analysis where CFR among treated children assumed to be half (3 per 10,000) and CFR among untreated children assumed to be double (1,000 and 400 per 10,000 for children <2 and 2-5 years old, respectively).

### Effective case management of hospitalized children with malaria

Seventeen studies were identified that reported hospital-based mortality data in children in zones of stable transmission in sub-Saharan Africa [64-80]. Wide variations were seen in quality of care offered, stringency of definition of severe malaria, and CFR (Additional File 1). When studies that offered moderate to high quality care and documented outcomes of all hospitalized children with a primary diagnosis of malaria were included, the weighted mean CFR was 3.4% (95% CI 1.6-5.2). When the definition was restricted to children with severe disease, the weighted mean CFR was 13.6% (95% CI 8.4-18.8). In the years immediately following the spread of chloroquine resistance (late 1980s), children who continued to be treated with chloroquine had approximately a three-fold higher CFR than children treated with an effective antimalarial [81]. Two studies were identified that reported hospital-based mortality data examining the impact of chloroquine resistance on mortality. CFRs in the setting of chloroquine failure were 13.1% (39/296) and 21.1% (500/2374) in two studies in hospitals that otherwise offered a high standard of care [67,82]. The resultant protective efficacy was 82%, with a sensitivity analysis range of 63-94% (Table 3).

**Table 3:** Estimates of protective efficacy of case management with intravenous quinine for children hospitalized with *P. falciparum* malaria

CFR range among children not receiving effective treatment	Estimated CFR range among children receiving intravenous quinine	PE range using CFR of 3.4% for treated and 13-21% for untreated	Midpoint PE of column 3	Lower bound	Upper bound
13.1%-21.1%	3.4% (95% CI 1.6-5.2)	76-87%	82%	63%	94%

CFR: case fatality ratio

Protective efficacy = (1-Relative risk) * 100, where Relative risk = CFR receiving intravenous quinine / CFR not receiving effective antimalarial

Lower bound based on sensitivity analysis where CFR among treated children is the upper bound for 95% confidence interval and CFR among untreated children is the lower of the two values for untreated children.

Upper bound based on sensitivity analysis where CFR among treated children is the lower bound for 95% confidence interval and CFR among untreated children is the higher of the two values for untreated children.

## Discussion

We performed systematic reviews to quantify estimates of PE for the following interventions for preventing mortality due directly to malaria in children in sub-Saharan Africa: prompt effective case management with ACTs for uncomplicated malaria, and effective case management with intravenous quinine of children hospitalized with malaria. The lack of controlled trials and the need to use numerous lower quality data sources make this exercise challenging.

Given the lack of placebo-controlled trials of treatment for malaria due to ethical reasons, we were unable to directly determine PE for prompt effective treatment of uncomplicated malaria for preventing malaria child mortality, as compared to no treatment. A meta-analysis was used to quantify the CFR for children with uncomplicated malaria that received prompt effective treatment. Results showed that death following uncomplicated malaria treated promptly with an effective antimalarial is very rare (0.068%, 95% CI 0.024 – 0.112). Because of a paucity of available data, we based the estimate of the natural mortality history of uncomplicated *P. falciparum* malaria on a previously published Delphi method, which found that experts estimated the risk of death among untreated children with uncomplicated *P. falciparum* malaria to be 5% for <2 year olds and 2% for 2-5 year olds. Combining these estimates, we estimated a very high PE of prompt ACT treatment of uncomplicated malaria compared to no treatment [99% (94-100%) for children younger than 2 years old, and 97% (86-99%) for children 2-5 years old].

For programmatic utility, we estimated a PE of effective case management of all children hospitalized with malaria. The CFR of 3.4% (95% CI 1.6-5.2) for all hospitalized children estimated by this method is very similar to that found by other authors [71,81,82], and the CFR of 13.6% for strictly defined malaria is slightly higher than the AQUAMAT trial [9], not surprising for observational studies compared to clinical trial conditions. As a proxy for mortality of untreated hospitalized malaria (natural history), we used mortality data from observational studies in the setting of chloroquine failure, during the era when chloroquine resistance was being established and chloroquine was still in widespread use. This resulted in the estimated CFR of untreated hospitalized malaria ranging from 13-21%. The higher bound (21%) is problematic, as the majority of the children in this sample who died had received chloroquine, and died despite respite receiving intravenous quinine after being admitted in critical condition, thus it may be a major underestimation. A qualitative study in Mali found that mortality for severe malaria (by maternal definition during interview) was 17.0% regardless of treatment-seeking [83], which falls in the middle of this range and lends some credence to its accuracy. Thus we believe that a PE of 82% for protective efficacy of effective case management with intravenous quinine is reasonable. Given that these studies all used intravenous quinine, the PE of intravenous artesunate for preventing mortality would be expected to be even higher.

There are numerous limitations to using the LiST model to predict the number of child deaths that could be prevented by scaling-up case management of uncomplicated and severe malaria. First, no placebo-controlled data exists and the historical literature is of insufficient quality to establish a CFR for children who go untreated for uncomplicated and severe *P. falciparum* malaria (natural history). For uncomplicated *P. falciparum* malaria, we relied on expert opinion from a Delphi survey to obtain an estimate of the probability of a child dying following uncomplicated *P. falciparum* malaria when left untreated, within the context of stable transmission. Such an estimate from a Delphi study is clearly subject to bias, although the direction and magnitude are unknown. For estimating the probability of a child dying from severe *P. falciparum* malaria when left untreated, we relied on a proxy estimated as the probability of a child dying from severe malaria after treatment with chloroquine, in the context of high chloroquine resistance, which ranged from 13.1-21.1%. While this was a helpful proxy, it likely underestimates the true untreated CFR and therefore underestimates the true PE of case management of children hospitalized with *P. falciparum* malaria. Second, while the majority of children with uncomplicated malaria are still not treated with an ACT, and many hospitalized children receive inadequate care, often with sub-optimal medication as well as ancillary treatment, many do receive some form of treatment. Thus hospitalized children who had received chloroquine likely had somewhat better outcomes than children who received no treatment. Third, our approach takes into account only a given episode of malaria, and does not take into account longitudinal impact of adequate or inadequate treatment, or the post-prophylactic effect of some antimalarials. If more reliable estimates of untreated mortality of uncomplicated and severe disease become available, it will be important to update the estimates. Finally, while it would be optimal to generate these estimates for children with stringently defined severe malaria, as CFR for severe malaria after treatment with intravenous artesunate has been well documented, no proxy for untreated mortality in severe disease currently exists. If these data become available, the protective efficacy of case management with intravenous artesunate in this subset of children should be estimated.

Case management coverage indicators in most sub-Saharan African countries lag far behind coverage indicators for malaria prevention, in part because the delivery of effective case management of both uncomplicated and severe disease is quite complex and involves not only procurement of the drugs, but supply chain management, health worker training and supervision, revision of payment schemes to remove financial barriers, extending this care to more peripheral levels of the health care system, and working to encourage use of these services by the population.

The currently recommended coverage indicator for prompt effective treatment is the proportion of children <5 years old with fever in the past two weeks who received an ACT within 24 hours from the onset of fever [84]. While an attempt has been made to standardize this indicator to be measured by most nationally-representative household surveys (e.g. Demographic and Health Survey, UNICEF’s Multiple Indicator Cluster Survey and Malaria Indicator Surveys), it is problematic for measuring trends in coverage over time for several reasons. First, as scale-up of prevention interventions such as ITNs increases, the proportion of fevers due to malaria is likely to decrease. Second, most countries have now adopted the policy of laboratory diagnosis [5], with either microscopy or rapid diagnostic tests, for all suspected malaria cases seeking treatment through the health system. For this reason when assessing trends in coverage for prompt effective treatment of malaria fevers, the proportion receiving treatment has actually gone down in most countries since 2005 as countries have switched from clinical diagnosis to laboratory diagnosis. Finally, most countries switched their first-line antimalarial from a monotherapy to an often less available ACT within the last 5-8 years [5], decreasing treatment with a recommended first line antimalarial in some countries over the past decade.

There is currently no established indicator for measuring the proportion of children hospitalized with *P. falciparum* malaria that receive effective case management. Establishing such an indicator will be difficult. Mortality of hospitalized patients depends not only on the quality of care received once hospitalized, but the delay in reaching such care, which depends on multiple factors including the distance to referral level care, availability of transportation, and cost of treatment, each of which may increase the delay and decrease the likelihood that the child will reach referral level care. However, programs are much more likely to be able to collect and report data on the management and outcomes of all children hospitalized with malaria than of a subset of children with stringently defined severe malaria.

## Conclusions

This systematic review attempted to quantify the PE of ACT used for treating uncomplicated malaria and effective case management with intravenous quinine for treating severe *P. falciparum* malaria for preventing malaria mortality in children <5. While there have been no placebo-controlled randomized trials of ACTs for uncomplicated disease and parenteral quinine or artemisinins for severe disease, our analysis shows these strategies to be a highly effective treatment in preventing malaria mortality in children, preventing nearly all (>95%) deaths that would result from uncomplicated *P. falciparum* malaria if left untreated, and over three-quarters (82%) of deaths in children with hospitalized *P. falciparum* malaria, compared to hospitalization without effective malaria treatment. Given standardized and valid coverage indicators can be developed, these PE estimates will allow the use of the LiST model for estimating the likely impact of past treatment scale-up, as well as for predicting future impact of treatment scale-up by national malaria control programs.

In this manuscript, we have provided our best effort at identifying methods to characterise the PE estimate for prompt effective malaria treatment for uncomplicated and severe malaria. We are now faced with the dilemma that there is currently no standardized indicator for both the true frequency of disease or access to these interventions in the population. While we welcome debate on the PE estimates we have generated, as well as future refinements, it is critical to develop standardized coverage indicators to measure progress, now and in the future. At this point, the coverage of good case management is low in most sub-Saharan African countries. In order to track progress as good case management coverage improves, we need standardized population-based estimates of the following: 1) proportion of children <5 with uncomplicated malaria receiving an ACT and receiving no treatment; and 2) the proportion of children <5 eligible for hospitalization with malaria receiving effective case management with intravenous quinine or artemisinin and receiving no treatment. It is imperative that the malaria community act now to establish these measures, test means of assessing them, and insert them into surveys and other population-based measurement tools that will allow tracking them over time.

## Competing interests

We declare no competing interests.

## Authors' contributions

JT and TPE performed systematic reviews, data abstraction, statistical analyses and drafting of the manuscript. RWS helped with drafting of the manuscript.

## Supplementary Material

Additional file 1 Web appendix reporting the characteristics of observational studies of case fatality rates in children hospitalized for malaria, broken into subgroups where multiple levels of severity or quality of care were reported Click here for file
